# What makes a perinatal woman suicidal? A grounded theory study

**DOI:** 10.1186/s12888-022-04015-w

**Published:** 2022-06-07

**Authors:** Holly E. Reid, Daniel Pratt, Dawn Edge, Anja Wittkowski

**Affiliations:** 1 Division of Psychology and Mental Health, School of Health Sciences, Faculty of Biology, Medicine and Health, University of Manchester, 2nd Floor Zochonis Building, Brunswick Street, Manchester, M13 9PL UK; 2 Manchester Academic Health Science Centre, M13 9NQ, Manchester, UK; 3 Greater Manchester Mental Health NHS Foundation Trust, Manchester, UK

**Keywords:** Women, Mothers, Perinatal, Suicide, Qualitative, Interviews

## Abstract

**Background:**

Suicide is a leading cause of maternal death during pregnancy and up to a year after birth. Psychological and psychosocial risk factors for maternal suicide ideation and behaviour have been identified but do not account for why mothers begin to experience suicidal thoughts. Qualitative research offers a way of identifying what might drive mothers to initially consider suicide and then go on to act on such thoughts; crucial for the development of assessments to identify, and interventions to target, maternal suicide ideation and behaviour. We aimed to develop a grounded theory outlining what makes women think about suicide and/or engage in suicidal behaviour during pregnancy and the first 12 months following birth?

**Method:**

Semi-structured interviews were conducted with 12 mothers in the UK who had suicidal thoughts during pregnancy and/or the first year following birth. A constructivist approach to grounded theory was adopted which guided the data collection and analysis processes.

**Results:**

We developed a model outlining the theorised process of psychological factors that culminates in mothers experiencing suicidal thoughts and then making a suicide attempt during the perinatal period. The process was initiated when mothers felt attacked by motherhood which led to feeling like a failure, self-identifying as a “bad mother” and subsequent appraisals of entrapment and/or defeat. When nothing resolved the distress and as mothers collated reasons for why they perceived they needed to die, suicidal behaviour became a viable and appealing option. We theorised that mothers might make a suicide attempt when they entered a state of intense “darkness” brought on by a trigger, followed by a temporary lapse in the conflict between the desire to live and desire to die and an opportunity to attempt.

**Conclusions:**

Participants stressed the rapid onset of suicidal thoughts. We suggest that healthcare professionals enquire about the mother’s feelings towards the baby and of isolation, how she views herself as a mother, feelings of entrapment and defeat during routine contacts to aid identification and prevention of suicidal ideation/behaviour. Suggested interventions to prevent suicidal thoughts and behaviour include helping women manage their expectations for pregnancy and the postpartum period.

## Introduction

In the UK and Ireland, maternal suicide is the leading direct cause of death between six weeks and a year after the end of pregnancy and the fourth largest cause of death during or within six weeks of the end of pregnancy [1]. Data for these maternal deaths also demonstrate that over the past decade, the rates of suicide during the perinatal period (i.e., pregnancy and the first postpartum year) have remained largely unchanged, with 0.55 deaths by suicide per 100,000 maternities reported in 2011–2013 [2] and 0.46 deaths by suicide per 100,000 maternities reported in 2017–2019 [1]. These rates highlight that current efforts to reduce maternal suicide during this period need to be improved. Losing a mother to suicide also has a profound effect on the child’s life, including increased risk of death by suicide themselves [3]. In addition, maternal suicidal ideation has been associated with poorer child cognitive outcomes, including motor skills and language development [4].

Identifying psychological and psychosocial risk factors for suicide is important for the future development of tools to detect and prevent or reduce suicidal ideation and behaviour. Previous quantitative studies have started to investigate a range of factors associated with an increased risk of suicide during the perinatal period, such as hopelessness and the impact of childhood trauma [5, 6]. In their systematic review, Reid et al. [7] identified 59 studies that investigated associations between psychological and psychosocial risk factors and suicide outcomes in pregnant and postpartum participants. Risk factors investigated by the 59 studies could be categorised as negative life events, social factors, cognitive factors, and personality and individual differences. The review reported strong evidence to indicate that experiencing adulthood and/or childhood abuse is associated with suicide ideation, attempts and death. Furthermore, a lack of social support was also found to be particularly important during the perinatal period and was significantly associated with suicidal behaviour. In their review, Reid et al. [7] also highlighted that many of the identified factors are not mutually exclusive; for example, experiencing negative life events can impact cognitive factors, such as worthlessness and shame. Although identifying these individual psychological and psychosocial risk factors offer a useful description of those most at risk of suicide, these factors alone do not explain why women begin to experience suicidal thoughts and then why some go on to act on those thoughts. Examining the interactions between these risk factors, and how they contribute to suicidal thoughts and behaviour, is crucial for the development of assessments to identify and interventions to target maternal suicide ideation and behaviour.

There are three predominant psychological theories of suicide that also account for the transition from having suicidal thoughts to acting on those thoughts. According to Joiner’s [8] *Interpersonal Theory of Suicide* (*ITS*), a desire for suicide occurs when there are both high levels of perceived burdensomeness (feeling a burden on others) and thwarted belongingness (feeling that you do not belong) [9]. An individual may act on this suicidal desire if they acquire the capability for suicide (reduced fear of death and increased tolerance for physical pain). The more recent *Integrated Motivational-Volitional (IMV) model* of suicidal behaviour [10] outlines a motivational phase whereby feelings of defeat and humiliation together with a sense of entrapment can result in the formation of suicidal ideation and intent. This model describes volitional moderators that govern the transition from suicidal ideation to suicidal behaviour, including exposure to suicide, impulsivity and fearlessness about death. Thirdly, Klonsky and May [11] propose the *Three-Step Theory of suicide* which hypothesises that a combination of being in pain and hopelessness leads to suicidal ideation. Individuals who also experience disrupted connectedness will have strong suicidal ideation and this will progress to a suicide attempt if the individual has the dispositional, acquired, and practical capacity to do so. Empirical research has demonstrated support for all three of these theories, although it has relied heavily on student samples [12–14]. These theories were also developed to account for suicidal ideation and behaviour in the general population but have not been tested specifically using pregnant and/or postpartum samples. The perinatal period brings unique changes and challenges for a mother to negotiate, such as breastfeeding, bonding with baby and the transition to motherhood [15]. This period has also been shown to stir up traumatic experiences involving women’s own parents and childhood experiences (also known as ‘ghosts in the nursery’ [16]) which could influence psychological and psychosocial factors. Therefore, we cannot infer that these theories of suicide are entirely applicable to perinatal women because these aforementioned factors, among others, specific to this period, are likely to be involved in suicidal ideation formation and behavioural enaction.

Qualitative research offers a way of identifying risk factors for maternal suicide and untangling the web of interconnected risk factors investigated previously in the perinatal population. In their thematic analysis with 14 North American perinatal women, who screened positive for current suicidal ideation, Tabb et al. [17] identified four themes which show that change in mood, negative experiences that the perinatal period can bring (e.g., miscarriage), mental health services, and situational coping, all have a role to play in maternal suicide. However, the authors did not explore the interplay between risk factors for suicide or the processes that could result in a perinatal woman feeling suicidal. In the only review of qualitative literature on this topic to date, Praetorius, Maxwell and Alam [18] conducted a meta-synthesis of eight qualitative studies, one of which was a grounded theory study, to investigate the lived experiences of mothers with postpartum depression who also experienced suicide ideation. Six themes were identified, which describe mothers hiding their suicidal feelings to adhere to the cultural expectations of motherhood, an incongruence between the expectations and reality of motherhood, loss of control, the overwhelm of motherhood on top of pre-birth responsibilities, lack of sleep, and social support as a buffer to suicidal thoughts. The authors noted that their findings bore similarities to Joiner’s *interpersonal theory of suicide* and elaborated on aspects of the theory to incorporate elements specific to mothers with postpartum depression. This included ‘thwarted motherhood’, which the authors propose is a subconstruct of ‘thwarted belongingness’ and relates to a mother’s lack of self-esteem because of her lost identity and feelings of incompetence when mothering. In the original *ITS*, liability and self-hatred are indicators of perceived burdensomeness. Praetorius, Maxwell and Alam [18] propose two additional aspects that contribute to liability and self-hatred: feeling like a ‘mommy failure’, and the ‘baby burden’ (unexpected challenges related to the baby that make motherhood more difficult, including breastfeeding difficulties and the baby’s temperament). The authors highlight that many aspects of the perinatal period can increase a woman’s physical pain tolerance which may contribute to a woman’s acquired capability for suicide. Praetorius, Maxwell and Alam [18] also suggested that the exhaustion associated with transitioning to becoming a mother contributes to the acquired capability for suicide although their description of why this is the case is unclear and does not account for those women who have older children, having previously experienced matrescence.

This reworking of an existing theory could be the first step in the development of a theory of suicide that incorporates experiences of perinatal women. However, reworking an existing theory may mean important factors and connections between these factors are missed. It is also important to highlight that Praetorius, Maxwell and Alam [18] meta-synthesis included qualitative studies that focused on women experiencing postpartum depression with suicidal feelings rather than primarily focusing on suicidal ideation and behaviour, irrespective of diagnosis. A grounded theory approach allows us to specifically explore how women may become suicidal and then go on to act on suicidal thoughts by establishing psychological and psychosocial risk and protective factors and theorising how these factors interact during the perinatal period. The previous research described that a web of interconnected factors, some unique to the perinatal period, may be involved in the formation of suicidal thoughts and progression to suicidal behaviour, and developing a model of these factors will prove useful for future tools to prevent suicide in this population. Thus, through the development of a grounded theory, we aimed to answer the following research question: what makes a woman think about suicide and/or engage in suicidal behaviour during pregnancy and the first 12 months following birth?

## Method

### Design

As suicidal ideation and behaviour in women during the perinatal period represent an under-theorised area, we aimed to develop a theory ‘grounded’ in the data and analysis. In contrast to other qualitative approaches, such as thematic analysis and interpretative phenomenological analysis, grounded theory [19, 20] allows for substantive theory development. More specifically, we adopted the constructivist approach to grounded theory which views research as constructed rather than discovered, with the resulting theory being grounded in both the participants’ and researchers’ experiences [21]. Constructivist grounded theory takes an ontologically relativist position, which posits that no single ‘true’ reality exists because reality is constructed within the human mind [22]. As researchers, relativism means we acknowledge that participants’ realities are contextually positioned within time, place and culture [23]. Constructivist grounded theory also assumes the epistemological position of subjectivism, which acknowledges that the researcher and participant will interpret the world in a way that makes sense to them; a useful approach for research that investigates how an individual’s experience shapes their perception of the world [22]. Charmaz [21] stated that constructivist grounded theorists can “investigate overt processes in painstaking detail and offer explanatory statements” (p. 259), which aligned with our aims of investigating and explaining the process that results in a mother experiencing suicidal ideation and/or behaviour during the perinatal period. Along with memo-writing, coding and sampling for theory development, Mills, Bonner [24] outline three requirements specific to constructivist grounded theory research: i) a sense of reciprocity between participant and researcher in the co-construction of meaning, ii) a participant-researcher relationship that attempts to modify any power imbalances, and iii) the researchers reflect on their own position and renders participants’ stories into theory.

### Ethical considerations

The study was reviewed by a National Health Service (NHS) Research Ethics Committee (18/NW/0849) and received ethical approval from the Health Research Authority. After giving informed consent, each participant provided their health professionals’ contact details (e.g., their Care Coordinator) in case it was identified during the interview that the participant required a health professional’s support. A distress management protocol was developed and followed by the researcher if a participant became upset or distressed during the interview or any contact with the researcher. On completion of the interview, all participants were provided with information for further mental health support and asked if they would like to receive a summary of the findings upon completion of the analysis.

### Participants

Women were included if they experienced suicidal thoughts, planned a suicide and/or attempted suicide during pregnancy and/or within the first 12 months following birth; this could have been at the time of recruitment to the study or in the past (there was no limit to how far in the past participants experienced suicidal ideation/behaviour). The focus was upon pregnancy and the first postpartum year given that suicide is the leading cause of death between six weeks and a year after the end of pregnancy and the fourth largest cause of death occurring during or within six weeks of the end of pregnancy [1]. Furthermore, specialist perinatal mental health services in the UK, such as inpatient Mother and Baby Units (MBUs) and Perinatal Community Mental Health Teams, currently only provide support to women during this period (i.e., during pregnancy and up to their child’s first birthday). Participants were also required to be 18 years or older at the time of consent. Women with limited ability to speak and understand English were excluded due to difficulties in obtaining informed consent and conducting interviews with a researcher whose first language is English. Women were not eligible to participate if they lacked capacity to consent or if they felt suicidal in the first 12 months following a termination, miscarriage, or stillbirth.

### Procedure

Participants were either referred to the study through an MBU, or self-referred through advertisements displayed in General Practitioner (GP) practices around Northwest England and posted on social media. All potential participants who enquired about participation were provided a participant information sheet. Once potential participants had read the information sheet, had all questions answered by the first author and remained willing to take part, they completed a consent form. The first author interviewed all participants and collected the demographic details and information regarding suicidal thoughts and behaviours via a demographic questionnaire. Although participants were asked to self-report any psychiatric diagnoses, prescribed medications when feeling suicidal, and their experiences of suicidal ideation, planning or attempted suicide; these details were not verified against official records for several reasons. Firstly, we trusted participants’ own understanding, knowledge and recall of their diagnoses, medication prescriptions and suicidal experiences. Secondly, a participant’s diagnosis and/or prescribed medication did not determine her eligibility to participate, rather this information was collected to help characterise our sample. Thirdly, most participants were recalling experiences in the past which would be difficult to verify using measures of suicidal ideation and behaviour. Similarly, many participants had not told anybody about their suicidal experiences or accessed support from services, and therefore verification of their suicidal experiences through their medical records or contact with their mental health professionals would not be possible, not to mention the element of distrust between the researcher and participant that this sort of verification could introduce.

Interviews were recorded using an encrypted Dictaphone and all interviews were transcribed verbatim, eight by the first author (HR) and four by a professional transcription service.

### Sampling

Instead of aiming to achieve ‘theoretical saturation’ whereby interviews no longer generate new properties of the pattern [25], we aimed for ‘theoretical sufficiency’ whereby adequate data are generated to construct a coherent model that answers the research question [26]. At first, initial purposive sampling was used whereby the first four women who met the eligibility criteria and returned consent forms were interviewed. Following this, theoretical sampling was used to seek participants that would challenge or extend ideas being constructed from the initial interviews [21]. For example, the initial participants all felt suicidal during their first pregnancy or after the birth of their first child, and so efforts were made to also recruit participants who experienced suicidal thoughts during the pregnancy or postpartum period with a child that was not their first. We then sought women who experienced suicidal thoughts during both pregnancy and the postpartum period rather than just during pregnancy or just during the postpartum period. Throughout recruitment, we made a particular effort to invite women from ethnically diverse communities to participate, to ensure adequate representation of mothers of different ethnicities, especially given maternal death is four-fold higher in mothers of Black ethnicity groups compared with white women [1].

### Interviews

We developed an initial semi-structured interview topic guide which was intentionally open and flexible to allow the initial participants to describe their experience of feeling suicidal at length and encourage participants to talk about anything pertinent to the research question. Through these detailed accounts we learnt of elements and ideas that could be explored in depth in future interviews, through modification of the interview guide. The guide was modified a total of three times; the first modification involved exploring the content of suicidal thoughts in more detail, inquiring as to what participants felt the ‘aim’ of suicide was and exploring participants’ feelings towards their partners and friends around the time the suicidal ideation began and while they experienced suicidal thoughts and behaviour. The second modification saw questions added to explore participants’ expectations and experiences of childbirth and their feelings towards work and motherhood. The third modification involved the addition of two questions to ascertain participants’ feelings towards their baby during pregnancy and whether breast/bottle feeding impacted their suicidal thoughts.

### Analysis

Data collection and analysis were conducted simultaneously with analysis beginning after the first interview. We followed Charmaz’s [21, 23] and Charmaz and Thornberg’s [27] guidance on conducting and ensuring quality in constructivist grounded theory studies. Firstly, HR became immersed in the data through either transcribing the interview recording verbatim, or when the interview was transcribed professionally, HR listened to the recording while reading the transcript which also served as a way of checking the transcript accuracy. She then read and re-read each transcript before coding. This immersion in the data is essential for researchers to begin exploring patterns and relationships, and to support the analytic imagination needed for theory generation [28]. Initial line-by-line coding involved reading each line of the transcript and inductively generating a code to account for that line if appropriate. Ideas could then be generated from the data by categorising segments of each transcript. Close attention was paid to verbs ending in ‘-ing’ (also known as gerunds) to capture actions and processes, metaphors and analogies which participants may use to represent their thoughts, behaviours and experiences and linguistic connectors, such as ‘because’ and ‘then’ which are used to describe associations [29]. Next, focused coding involved selecting the most prevalent and important codes to produce a set of central codes, often these were codes that were repeated within a transcript and across transcripts [29]. Finally, theoretical coding involved refining the final categories in the theory and relating them to one another which was helped by the researchers’ abduction (i.e., their own pre-existing ideas, experiences and knowledge of relevant literature) [30]. Throughout this process the constant comparative method was used, an essential part of grounded theory methodology [31]. This method involves the continual comparison of data between and within interviews, it is then used to compare codes to one another and to the data from which they are developed. Finally, the developing theory is also subject to regular comparison to the interview data, the codes and the previous theoretical ideas or diagrams.

**Fig. 1:**
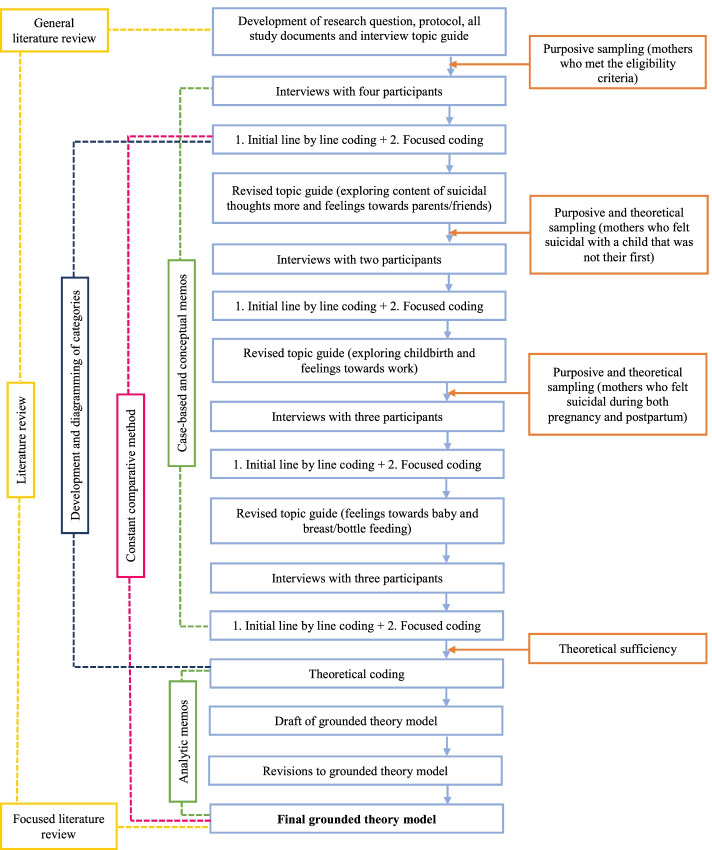
Flow diagram to demonstrate this study’s grounded theory methodology

The first author kept case-based memos after each interview to reflect on her impressions of the participant’s experiences and what she learnt during the interview. Conceptual memos were also written to compare data, cases and codes, record thoughts on the direction of the analysis and raise questions to ask in future interviews. Member checking of the participants’ representations of their experience of suicidal thoughts and/or behaviour occurred during the interviews, whereby the interviewer ‘played back’ aspects of their understanding of the experience to the participant to ensure accuracy and check for misinterpretation [32]. Throughout the data collection and analytic process, diagramming was also used to combine what was captured in the conceptual memos and the developing codes. Diagramming is a useful tool to synthesise the data and facilitate theory development by grounded theorists [33]. See Fig. 1 for a diagram outlining the data collection and analytic process used in this grounded theory.

### Reflexivity

Reflexivity sees the researchers consider their position and influence on the research, which is particularly important when conducting a constructivist grounded theory because the researchers are a part of the construction rather than objective onlookers [24]. HR, a PhD student, conducted the latter four interviews while pregnant with her first child and completed the final stages of analysis after the birth of her first child; this meant HR made sense of participants’ narratives within the context of her own transition to motherhood. HR also had previous experience of interviewing mothers and fathers during pregnancy and the postpartum period (e.g., [34, 35]. DP is a senior clinical lecturer, clinical psychologist, and father with expertise in the development and evaluation of suicide prevention interventions. DP has extensive clinical experience of delivering, and supervising the delivery of, psychological therapy to individuals experiencing suicidal ideation and/or behaviour across community, mental health inpatient and forensic settings. DE is a professor of mental health and inclusivity, a mother, and experienced health services researcher with expertise in qualitative methods and developing psychosocial interventions. AW, a senior lecturer, clinical psychologist, and mother, has extensive clinical and research experience of and expertise in perinatal mental health including qualitative research. Through her clinical work on an inpatient MBU, AW has direct experience of women trying to cope with suicidal ideation and behaviours. Reed and Procter [36] propose that researchers can occupy different positions with regards to their relationship with the research environment. We believe, as researchers who undertook this research in an area that was familiar to us, yet we were not researching a phenomenon that any of us had experienced ourselves, that we mostly occupied a ‘hybrid’ position. Because of HR’s hybrid position and her principal role in the study design, data collection and analysis, she chose to avoid reading literature around other models of suicide and perinatal mental health difficulties, while designing the study, collecting and analysing the data. This approach was taken to exercise control over what was added to HR’s already broad knowledge base of the subject area and limit how much other related models influenced her approach to and interpretation of the data. All authors met regularly to discuss sampling, recruitment methods, modification of the topic guide, coding, and theory development. Bridling describes the act of reflecting on one’s own presumptions and slowing down to find alternative interpretations of the data [31, 37]. Our efforts to bridle involved regular team meetings; the diversity within our author team with regards to clinical background, parenting experience and experience of conducting qualitative research, allowed us to challenge each other’s presumptions and pre-conceptions when collecting and analysing the data. Additionally, different team members suggested theoretical sampling directions to challenge data interpretation and the developing theory. Our collective personal parenting experience and research expertise with different parent groups gave us an understanding of how parenthood is experienced without suicidal feelings involved. This understanding will have influenced the analysis process, ensuring we focused on the phenomena of suicidal ideation and behaviour within the context of the transition to motherhood.

## Results

### Participant characteristics

Between March 2019 and January 2020, 13 participants were recruited and 12 were interviewed. One participant withdrew consent before being interviewed due to personal circumstances that made participation too difficult. It should be noted that all interviews were conducted prior to the outbreak of Covid-19 in the UK and associated restrictions being imposed in March 2020. Interviews ranged in length from 32 minutes to 1 hour 27 minutes.

Participant characteristics are outlined in Table 1. The age of participants at the time of the interview ranged from 27 to 47 years (mean 36 years), most were married or living with their partner, and were University-educated. Ten participants were White British, one was Arabian, and one was White Eastern European. Two participants reported feeling suicidal during pregnancy only, three felt suicidal during pregnancy and the postpartum period and seven felt suicidal during the postpartum period only. None of the participants reported any social services involvement.

**Table 1:** Participants’ demographic information and suicidal experiences

Pseudonym	Gestation or infant’s age at onset of suicidal thoughts	No. of children at onset of suicidal thoughts	Psychiatric diagnosis received from healthcare professional (if applicable)	Specialist perinatal support accessed (if applicable)	Suicidal thoughts	Suicidal planning	Attempted suicide
**Suicidal when pregnant**							
Pregnant mother 1	6–8 weeks gestation	0	Recurrent depressive disorder	Perinatal outpatient service	✓	✓	✓
Pregnant mother 2	9 weeks gestation	0	Obsessive compulsive disorder	PCMHT	✓	✓	
**Suicidal when pregnant and postpartum**							
Pregnant and postpartum mother 1	36 weeks gestation (in pregnancy) 3 months old (postpartum)	0 (in pregnancy) 1 (postpartum)	No diagnosis received	Perinatal wellbeing group	✓		
Pregnant and postpartum mother 2	8 weeks gestation (in pregnancy) 8 months old (postpartum)	0 (in pregnancy) 1 (postpartum)	Depression	None	✓		
Pregnant and postpartum mother 3	20 weeks gestation (in pregnancy) Newborn (postpartum)	0 (in pregnancy) 1 (postpartum)	Depression	None	✓		
**Suicidal when postpartum**							
Postpartum mother 1	3 weeks old	1	Severe postnatal depression	MBU	✓		
Postpartum mother 2	6.5 months old	1	Generalised anxiety disorder	MBU	✓	✓	✓
Postpartum mother 3	4 months old	1	Postpartum psychosis	MBU	✓		
Postpartum mother 4	4 months old	2	Depression and anxiety	None	✓		
Postpartum mother 5	Newborn	1	No diagnosis received	PCMHT	✓	✓	
Postpartum mother 6	1 week old	1	Atypical anorexia	None	✓	✓	
Postpartum mother 7	Newborn	1	No diagnosis received	None	✓	✓	

*MBU* Inpatient mother and baby unit, *PCMHT* Perinatal community mental health team

### Overview of qualitative findings and model

**Fig. 2:**
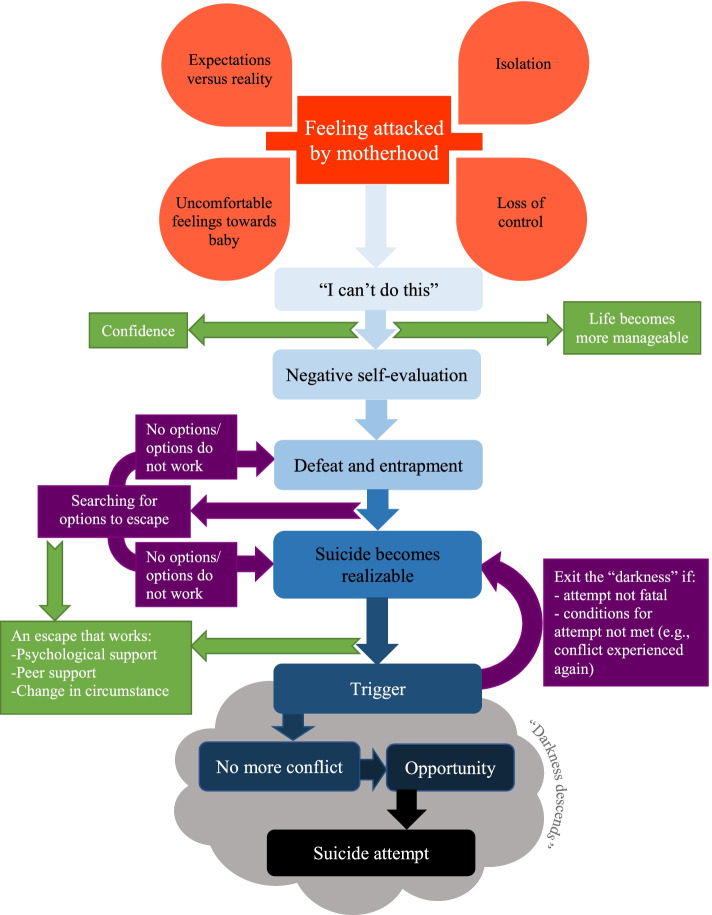
The grounded theory model of suicidal ideation and behaviour development during the perinatal period

Figure 2 outlines a visual representation of theoretical model ground in the data. The model outlines the theorised process that culminates in a mother experiencing suicidal thoughts and making a suicide attempt during the perinatal period. Our sample included women who felt suicidal during pregnancy only, women who felt suicidal when postpartum only and women who felt suicidal during pregnancy and postpartum, and this model was developed to ensure it is applicable to all three groups of mothers. The first element of the model outlines how feeling attacked by motherhood was central to all mothers’ experiences and triggered the cascade of feelings and thoughts in this process. Participants cited feelings and experiences of isolation, loss of control, not meeting their expectations for motherhood and having uncomfortable feelings towards their infants, which contributed to feeling attacked by motherhood. Women would then feel like a failure in their perceived inability to mother (i.e., fulfilling all the baby’s needs as the primary caregiver, having responsibility for the baby’s wellbeing, while feeling connected to the baby) and this led women to feel like a “bad mother”. Some postpartum participants reported trying to resolve these feelings by avoiding mothering, by staying in bed or delaying attending to their baby, but these efforts proved ineffective at changing negative feelings. The realisation that nothing changed these negative feelings and that the initial overwhelm was still present, led to mothers feeling trapped, and some felt defeated. This entrapment and defeat led women to conclude that the only option to escape was through suicide, thereby triggering suicidal ideation. Mothers reported feeling that others would be better off if they were not here, which turned these initial suicidal thoughts into a necessity rather than an option for the mother and perceived by the mother as beneficial for others. When suicide was viewed as the only option and this option became more necessary and hence appealing, acting on these thoughts became a real possibility. The model also theorises a subsequent transitional process for how a mother might move from experiencing suicidal thoughts to making a suicide attempt. Mothers often talked about entering a state of “darkness” whereby their suicidal ideation became intense and immediate, and this was brought on by a trigger (such as an argument with their partner) and a temporary lapse in the conflicting feelings around suicide (such as stopping worrying about who might find their body or whether the attempt would end in a fatal outcome). Mothers’ narratives revealed that they then had to have the opportunity which involved having access to means and knowing the baby would be safe. Once these three elements were fulfilled during the time of “intense darkness”, the likelihood of a mother making a suicide attempt was described as being very high. The speed at which this theorised process occurred varied across mothers but some highlighted that it could be very rapid.

### Feeling attacked by motherhood

Feeling bombarded was central to each mother’s narrative and commenced prior to her suicidal thoughts. Exactly what each mother felt attacked by varied in content, but it was always related to their new reality as a mother or the prospect of becoming a mother:

*“It would be such a big change for me having a baby I was like ‘think of all the stuff you’re going to lose, your kind of freedom […]* *you can’t plan your career, you don’t even know if you’ll be a good parent’, all that was kind of getting a bit much”.* (Pregnant mother 1).

Many mothers’ narratives described feeling as though they faced a barrage of external issues that came because of, or were exacerbated by, pregnancy/motherhood:

*“I didn’t have my Mum and Dad there, I didn’t- I still don’t have enough friends […] I think it was being attacked by lots of things around me”.* (Postpartum mother 2).

Other mothers’ experiences were more internal, whereby they struggled with a deluge of thoughts, feelings, or emotions:

*“I also felt overwhelmed, it was so many conflicting emotions, I know some people look at it and say ‘oh well that’s just your hormones’, but no it was in my head, it was in my thoughts […] it feels overwhelming and that’s how I felt at that point, all the emotions I was going through when they were strong. I didn’t know what to do with them or what sort of box to put them in”.* (Pregnant and postpartum mother 1).

The feeling of attack could stem from one major cause or a multitude of causes and although the cause(s) varied between mothers, we could identify four common ‘issues’ that contributed to mothers’ bombardment: loss of control, isolation, expectations versus reality and uncomfortable feelings towards the baby. We describe each of these ‘issues’ in more detail in the following subcategories 1.1, 1.2, 1.3 and 1.4. Postpartum mother 3 summed up some of these contributing ‘issues’ when she described her feelings once she had had her baby:

*“I felt out of control of my emotions, I felt scared, I felt alone, I felt very conscious that I was having a very different start to motherhood, to what I sort of thought I would have. And what I thought the people around me were having”.* (Postpartum mother 3).

### Loss of control

Being pregnant or having a baby resulted in a degree of relinquishing control and often this was a stark contrast to mothers’ lives prior to their pregnancy or postpartum: “*I like to be in control of things and I think I didn’t have any of that”* (Postpartum mother 5). Often mothers described being in control as an essential part of their work-life, and this newly experienced loss of control was something some mothers found difficult: *“I’m such a spreadsheet kind of person, I just couldn’t abide by the randomness of it all”* (Postpartum mother 1) and *“I remember my partner’s mum saying ‘ … motherhood isn’t something you can plan... she’ll do what she’s gonna do’. And I just, I think I thought ‘but I need to plan, I need to understand’. I felt like I didn’t know what I was doing”* (Postpartum mother 3). Other women felt disempowered, for example, during birth. Perceiving their birth as traumatic was often cited as a contributary factor to their negative feelings postpartum and we identified a feeling of powerlessness as a common factor in their birth experiences. Postpartum mother 6 described powerlessness over multiple elements of her birth; inability to help her baby while in distress, over those present at her birth and consequently over her dignity:

*“Because [the baby] was in distress now as well and at that point the room was full with like doctors, midwives, nurses […] you’ve got overwhelmed with pain, them looking at you down there sort of thing […] it was so undignified and just so- I just felt awful […] I was laying there thinking ‘oh my God, what have I done?’”.* (Postpartum mother 6).

Mothers also described that they had lost control of their environment and being pregnant or having a baby exacerbated this lack of control. This environmental change was always in relation to an inadequate home situation or living arrangements:

*“We had moved into a flat that had no furniture, no carpet, and we’d moved when she was two weeks old […] and the washing machine broke down […] and I just lost the plot […] it’s those things out of your control. There was so much that was out of my control”.* (Postpartum mother 7).

### Isolation

All mothers reported an experience of feeling isolated. Some participants spoke about how changes brought about by pregnancy or by the arrival of their baby resulted in a change in their relationship with their partner and this often led mothers to feel isolated from the person they were closest to. Pregnant and postpartum mother 2 described her partner’s reaction when she told her partner she was pregnant:

*“He immediately went back to sleep. And I thought ‘fuck, this is what I’m dealing with, I’m on my own in this’ and I think I very much, right from the beginning, thought, ‘he’s just like not interested’”.* (Pregnant and postpartum mother 2).

This feeling of being alone during the perinatal period, extended to situations with other mothers and babies. Mothers described how they felt other mothers had stronger support networks and ‘knew what they were doing’; this could lead to mothers questioning their suitability to parenting:

*“I remember thinking very early on after I’d had [baby] and we were all out with our babies I was like ‘am I doing it wrong? How they are with their babies, should I be like that with my baby?’ so I think I only ever saw them [other mothers and babies] from this perception of being judged”.* (Pregnant and postpartum mother 2).

We also found there was a cyclic relationship between the overwhelm and mothers’ isolation. Mothers found themselves avoiding talking to friends and family when they were finding their pregnancy or the postpartum period difficult. Participants feared others would identify there was something wrong or they might be judged during contact with others:

*“I was just getting really, really stressed but at the same time cutting myself off from people because I didn’t want people to ask, ‘what’s up with you?’, ‘what’s going on?’ because then I’d have to explain so I was getting more isolated”.* (Pregnant mother 1).

### Expectations versus reality

It became apparent that participants had certain expectations for the perinatal period, such as expecting to meet their breastfeeding goals, having a drug-free birth, and having a loving relationship with their partner. However, many mothers found their reality did not meet these expectations, and this added to their feeling of distress. Pregnant and postpartum mother 3 described how, although she was married, her partner was not involved in her life when she was pregnant or after having the baby, which was at odds with her expectations:

*“In my head there was always this idea that if, you know, you’re married and you’re pregnant, you’d have a partner […] I guess I felt like I was sort of like a single married part-mum, or pregnant mum and in my head it didn’t- I never expected it to be like that”.* (Pregnant and postpartum mother 3).

Mothers also described societal expectations that they felt they had to meet, particularly that motherhood was supposed to be the happiest time of her life. This perception added another pressure and fear when mothers did not feel this way and ultimately contributed to feeling attacked:

*“I’m supposed to feel happy and, you know, the whole expectations where we’ve waited so long to have a child due to fertility treatment, that I felt guilty and that I wasn’t worthy of being a mum because of the pressures society puts on you”.* (Pregnant and postpartum mother 1).

Although most women described huge discrepancies between their expectations and reality during the perinatal period, participants never described anything or anyone that helped manage their expectations or made them feel more prepared for the reality they faced, despite many participants attending antenatal classes.

### Uncomfortable feelings towards the baby

All mothers spoke about having feelings towards their baby that they perceived as negative, and they believed that this was not the way they were supposed to feel; we have termed these ‘uncomfortable feelings’. Some mothers reported feeling “nothing” towards their baby, resentment towards their baby, not wanting the baby or an intense fear of harming their baby:

*“I didn’t love it [the baby], I didn’t wanna be with it, but if I wasn’t with it, I’d worry what would happen to it, so everything about that baby caused stress”.* (Postpartum mother 1).

These uncomfortable feelings were not only experienced by women who had had their babies, but they were felt by pregnant women too: *“I had negative feelings often about the baby as being like a parasite rather than this lovely thing”* (Pregnant and postpartum mother 2).

We noticed many mothers expressed emotional distance from their baby by referring to their babies as ‘it’ or ‘the baby’ rather than by their name or ‘my baby/son/daughter’. The entrenchment of these negative feelings towards their babies in the language mothers used demonstrates how strong the lack of connection was and, subsequently, how distressed this could make mothers feel.

Often mothers described how they had been expecting to feel a powerful and instant rush of love for their baby immediately after the birth and when participants did not experience this, they perceived this as abnormal. Participants personalised this abnormality, assuming it must be something wrong in themselves, and it often initiated the uncomfortable feelings towards their baby:

*“I didn’t get that instant bond […] other people talk about like that instant lightening bolt sort of thing. And because I was expecting that, and then I felt bad because I hadn’t got that […] I just felt completely overwhelmed”.* (Postpartum mother 6).

Some women also highlighted that they felt their connection with the baby was lacking: “*I loved the baby, I cared for the baby, but there wasn’t a bond with the baby. It was like, you know, having a baby doll”* (Pregnant and postpartum mother 3)*.* This distinction between loving and bonding with the baby was something a few mothers identified which is interesting because most of the participants were mothers for the first-time, but they still had a perception that having a bond with the baby was an additional but necessary feeling. These uncomfortable feelings could make caring for the baby difficult: *“I didn’t want to touch her, I didn’t want to be around her, I didn’t want to hold her”* (Postpartum mother 3). Caring for a new baby is all-consuming and challenging for all mothers, but when one is required to do this when feeling discomfort towards the baby this could exacerbate the relentless and unappreciated nature of caregiving.

Ultimately, we theorised that the loss of control, isolation, discrepancy between their expectations and reality, and uncomfortable feelings towards the baby all fed into feeling attacked by motherhood whereby participants perceived the world as a mother as being ‘too much’ to negotiate.

### “I can’t do this”

Once mothers felt attacked, they started to process this feeling of bombardment – how they felt about feeling this way and what it meant for them. All mothers said that they very quickly reached a point after feeling attacked where they were not able to ‘do this’ and it was striking how many participants used exactly the same phrase to describe this feeling, for example: *“I can’t do this, I can’t do this, this is deep”* (Postpartum mother 1) and *“I just felt like I can’t do this”* (Postpartum mother 6). We interpret this perceived inability in two ways: mothers evaluating themselves as failing in their mothering abilities and, secondly, not feeling able to cope.

Some mothers spoke about their feelings of failure in response to the aforementioned motherhood expectations. This feeling of failure could also be compounded by comparing themselves to others who they perceived to be ‘succeeding’. A common source of perceived failure was an inability to meet their breastfeeding goals:

*“So when I had [baby] everybody was very ‘earth-mothery’ and they all- all their babies breastfed and because mine wouldn’t or couldn’t I was like ‘oh god I’ve failed at another thing, like I couldn’t do the proper birth and now I can’t do the proper breastfeeding, like why can’t I do these things’ in my head, like, ‘why can’t I do what proper mums do?’”.* (Pregnant and postpartum mother 2).

This sense of failing seemed to spiral for mothers and started to become a common way for mothers to respond to situations:

“*… when the midwife came in and I had a hat on him in the house and she like whipped his hat off and said something about ‘he doesn’t need a hat on in the house, you’ll overheat him’, I thought ‘oh my God, I can’t even do that right'”.* (Postpartum mother 6).

The perinatal period presents a time of intense learning to care for a (nother) child and many mothers received differing advice from different sources, which could exacerbate their uncertainty, lack of confidence when mothering, frequently leading to them feeling failure. Therefore, having more confidence in mothering, which could also lead to fewer or no negative comparisons with other mothers, may protect a woman from progressing our theorised process and developing suicidal ideation/behaviour.

Secondly, mothers exclaimed “I can’t do this” in relation to feeling unable to cope and continue as they were. We theorised that this stemmed from feeling attacked over a sustained period and/or a lack of coping strategies to navigate this. This was also the start of mothers feeling as though something had to change or end because their situation was unmanageable:

*“I wasn’t coping with something in particular, or as well as I thought I should, and then that would start me thinking ‘well I can’t do this anymore’, you know it’s ‘I’m not helping anybody, I’m just making a mess’ or ‘making things worse’”.* (Postpartum mother 4).

Like the sense of failure, this feeling of being unable to cope managed to infiltrate many aspects of the mother’s everyday life, which demonstrates how easily an initial thought or feeling could spiral and affect how these mothers perceived situations around them.

We theorised that isolation could be a major driver for the feelings of failure and inability to cope. This is due to the lack of respite from the issues causing the feeling of being attacked during this challenging and unchartered period for mothers. It is also at this point when participants started to feel as though their experience was not the same as it was for other mothers, and concern about the finality of having a baby set in: “*I just thought, ‘I’ve made a terrible mistake, I can’t do this. This is all wrong, I’m not supposed to be feeling like this, this is all wrong’”.* (Postpartum mother 7). Therefore, we hypothesise that accessing things that make her life more manageable, could buffer against this sense of not coping and lead to a mother exiting the process leading to suicidal ideation/behaviour. These could include receiving help with childcare, having a supportive partner and contact with other mothers who may be struggling. We theorised that both the perceived failure and inability to cope can result in a mother quickly progressing to the next stage in our process and negatively evaluate themselves as mothers. Whereas, only feeling either failure or an inability to manage, may slow down this progression.

### Negative self-evaluation

The feeling that mothers would be or were a “bad mother” was central to mothers’ narratives and we theorised that this negative self-evaluation was necessary in driving a mother’s suicidal desire by lowering her own self-worth:

*“‘I’ll be a terrible mother’ and ‘my relationship’s awful’ and that sort of spiralling thought pattern of ‘actually everything’s getting worse and worse and worse’ […] I got pregnant in the October and probably by the January/February I don’t think I was even able to recognise myself”.* (Pregnant and postpartum mother 2).

As this negative self-evaluation emerged as an incredibly important part of women’s experiences of feeling suicidal, we interrogated this idea of being a “bad mother” in later interviews. It appeared that the shame and guilt associated with being a “bad mother”, along with the intense fear of not giving the baby a ‘good life’ gave this negative evaluation weight in participants’ experiences:

*“I’m going to be a rubbish mum […] my baby will just have a horrible life and will absolutely resent me because I’m this rubbish person [...] My idea of a rubbish mum is somebody neglecting their child […] there was a comment made by a professional that we like all pregnant ladies to be calm because of the cortisol levels. That could trigger your child to be exposed to mental illness. So, I’m thinking ‘oh, I’m already depressed, I feel anxious, I’ve got OCD, oh my child’s going to be born with all these symptoms and- so neglecting a little baby’”.* (Pregnant mother 2).

This participant demonstrates how exposed, and vulnerable to judgement by others, mothers can feel. Pregnancy and motherhood can be expected to involve providing limitless care and protection to the baby and when mothers felt unable to do this, it led them to feel as though they were not fulfilling their main purpose and to fear that they were impacting their child negatively.

We also identified an element of finality associated with being a “bad mother”, whereby mothers were assigning themselves this trait: *“I’ve not got this bond, so I’m a bad mum, I’m not going to be able to be a good mum to him. And I just- I felt like that was never ending”* (Postpartum mother 6). It became apparent that mothers did not feel they could become a ‘good mother’ and therefore this negative self-evaluation was fixed and became integrated into their identity. As evident in this quote, mothers also used those factors that contributed to the initial feeling of being attacked to come to their evaluation, for example, struggling to bond with the baby. Each participant acknowledged that they were never told they were a “bad mother”, it was always a conclusion arrived at by the mother herself.

Ultimately, being a “bad mother” was deemed worse than dying and therefore this step in the process was crucial for driving suicidal desire: *“I thought I’d rather [the baby] wasn’t here than had a mum who wasn’t sure if she wanted it or couldn’t do it, that to me would have been worse than killing myself”* (Pregnant mother 1). This participant introduced the idea that dying or not having the baby was a better situation than being a mother who could not fulfil her mothering purpose: to want the baby and be able to mother. We theorised that the importance of being a ‘good mother’ was ingrained in most women from modelling their own mother’s behaviour, to aspiring to be the mother society expects, to an inherent evolutionary need to ensure the prosperity of their offspring. Therefore, when a mother felt like she could not be that and she could not change this negative self-evaluation, the feelings of needing to escape emerged.

### Defeat and entrapment

Participants consistently reported feeling a need to escape because they felt there was no obvious way to resolve their distress: *“I just couldn’t see a way out”* (Pregnant mother 1). This inability to alleviate the bombardment, failure, inability to cope and negative self-evaluation led mothers to feeling trapped and/or defeated by their situation. The finality of having a baby seemed to be the main concern for mothers when assessing their options for change:

*“What I really wanted to do was to rewind to before, but what- I just couldn’t […] And so, I suppose I just went through this [thought of] ‘my life is unbearable therefore I have to die because I can’t bear my life’”.* (Postpartum mother 7).

This participant described wanting to return to her pre-baby life, and many mothers contemplated different options to alleviate how they felt. When participants came to the realisation that there was no solution, this was the point at which dying, the baby dying or both dying were the only perceivable options available, therefore suicide became a viable option thus triggering suicide ideation. Postpartum mother 1 expressed both feeling defeated and trapped by her situation:

*“I was like ‘uhhhh who can be bothered with this? Who wants to be changing pooey nappies in the middle of the night?’ and then it turned from not liking it to absolutely resenting it […] and I was like, ‘there is no way to make this any better, that’s it, this is my life now, there’s no way, like could I get him adopted out’ […] my life has been trying to solve problems and I’m like, ‘there’s no solution to this. Either he dies or I die’”. (Postpartum mother 1).*

The idea of offering the baby for adoption was only mentioned by a few mothers and this option was considered and then rejected very quickly, in favour of suicide. We theorised that the shame and emotional pain involved in having the baby adopted was worse than dying for these participants. Postpartum mother 1 also demonstrated how, for many mothers, the inability to solve a problem was a completely new feeling, at odds with their pre-baby lives which made women feel even more out of their depth. Mothers voiced their defeat through questioning why they wanted to keep going in this current situation, for example: “*just thinking what is the point and thinking this feels like this is forever and I don’t see an end to it”* (Postpartum mother 3). Other participants emphasised the entrapment resulting from the initial feeling of being attacked: *“it’s extremely hard for me to get out of the bed every single day because the thoughts keep attacking all the time no matter what I do”* (Postpartum mother 2)*.* Rather than lacking motivation or the ability to battle on which is more typical of defeat, Postpartum mother 2 described how she was willing to continue because she had tried to do different things to avoid or lessen her thoughts, but this had not worked. Defeat and entrapment are closely linked appraisals, but it was unclear as to why some mothers felt more defeated by their situation and others more trapped.

Women described experiencing passive suicidal ideation initially, rather than thoughts explicitly of death, women described the thoughts as wanting to: *“just kind of opt-out of your own brain”* (Pregnant and postpartum mother 2) and *“I just wanted to run away, not commit suicide as such, but just disappear”* (Pregnant and postpartum mother 1). These examples suggest that the primary aim of suicide for mothers was not to die, per se, but to alleviate how motherhood and their circumstances were making them feel. It was only in the later stages of this process that the suicidal ideation became more intense, with more of a focus on how to attempt suicide and the logistics around dying (including who would find the body).

At this point in the process, some postpartum mothers described that once they felt defeated and/or trapped they tried to avoid their distress: *“I basically dealt with it by just trying to sleep as much as I could, and not wanting to get out of bed”* (Pregnant and postpartum mother 1). Mothers were not explicit as to what the aim of this avoidance was, but it appears to be an attempt to alleviate the negative feelings, such as failure, that had arisen from mothering. However, this avoidance was only temporary because the baby would need attention eventually and this strategy proved ineffective at helping women escape their distress.

### Suicide becomes realizable

Once suicide becomes an option for relieving their feelings, mothers started to feel that suicide became more necessary for resolving their distress: *“[suicide] was starting to get more and more appealing the more I went round in circles about what to do”* (Pregnant mother 1). The development of suicidal ideation after the initial thought was not necessarily a linear process; mothers still searched for other options to escape the way they felt, but as time passed and these alternative options did not arise, suicide became a more viable option, and the ideation became harder to resist.

It was at this stage that mothers started to collate the reasons for dying by suicide. The major benefit that mothers believed suicide would bring was that it would make life better for those close to her: *“[baby] would be better off without me and [partner] will be better off without me and I can’t believe what I’m putting people through”* (Postpartum mother 3). Participants did not provide any examples as to how their families would benefit from them dying, it was a perception primarily born from the negative self-evaluation and again it was a conclusion arrived at by the mother rather than from any external evidence.

As a result of thinking through the reasons to die, women described the suicidal pull becoming stronger. However, mothers also described feeling an internal conflict because they also had a will to live:

*“It was a very, very fractured mind […] there was a rational but quiet bit going ‘I don’t want to die’. But it got drowned out by the very, very, very strong suicidal suggestions which just wouldn’t go away”.* (Postpartum mother 7).

This description gives a clear insight into how the suicidal desire can rapidly overpower the inherent desire to live. This idea that the mother’s mind was in two separate parts pulling her in different directions, and the relentlessness of this, must be emotionally exhausting to endure. It also presents another problem that needs resolving on top of the other ‘problems’ in the process that have gone unresolved and led to this point. This visualisation of two powers conflicting to influence a mother’s decision to die by suicide was used by another mother to describe how she experienced this stronger suicidal ideation:

*“it’s like having an argument with yourself, it is like having- you know, a devil on each shoulder kind of thing, where you’ve got one trying to be rational and the other one saying ‘oh just do it, why not, get it over with’ […] and the other one kicks in and is like ‘yeah but what about the kids’  you know, the kids will do something small or your hubby will give you a hug or something like that and you think ‘oh yeah that’s the thing that you’re here for’”.* (Postpartum mother 4).

This quotation highlights how complex this period is for mothers, because the changes brought about by the pregnancy or having a baby can trigger the start of this process leading to suicidal ideation and behaviour, but it can be the baby or family unit which can reduce that suicidal desire.

It seems at this point mothers really scrutinise the particulars of attempting suicide but even after consideration of issues and worries, such as the attempt not resulting in a fatal outcome, mothers often still perceived suicide as the only solution.

*“[suicide] literally seemed like that was the only way out of the situation. Like whether it worked or not, whether, you know, faith or not being able to have the courage to carry it out or it was going to be painful, it literally seemed like- honestly now I can’t comprehend it, but at the time it sounded like the most logical thing to do, the only thing to do […] it was the only way out”.* (Pregnant and postpartum mother 3).

This quotation demonstrates just how desperate mothers felt because the issues around dying by suicide seemed less important to them when compared with the need to escape. It also highlights the level of consideration that mothers undertake before feeling they only have one option, and this contradicts the idea that suicide is an impulsive decision.

We feel it is important to add here that the sample mainly comprised of mothers who did not attempt suicide and we theorised it is at this stage in the process that most of our sample were relieved from the suicidal pull and escaped this process. Mothers described how psychological support: *“mostly CBT[…] mainly sort of challenging my thoughts around why I thought I was a bad mum and why that wasn’t true”* (Postpartum mother 5), and peer support: *“mixing with other mums that weren’t- they were just still normal people, they didn’t get things right all the time”* (Pregnant and postpartum mother 2) helped to alleviate their negative thoughts and feelings, particularly negative self-evaluation, which then helped them escape this process. One mother also stressed how a change in circumstances helped her:

*“Moving out of the house I was in, where my friend had [mental health and substance misuse issues], as soon as I moved out, I got a different perspective on- ‘the world’s kind of ok outside this bubble you’ve been living in’. That helped loads”.* (Pregnant mother 1).

These mothers were then able to recover from their suicidal feelings. Mothers who do go on to act on their thoughts may not receive any support to alleviate the thoughts and feelings at previous stages in our theorised process, and/or may experience this stage (*Suicide becomes realizable*) more intensely whereby the inherent will to live is eventually overpowered.

### Moving from suicidal thoughts to suicide attempt

Only two women interviewed had acted on their thoughts of suicide. However, all participants were asked what they felt moved a woman from thinking about suicide to making a suicide attempt. It became apparent that mothers experienced two types of suicidal thinking: those that were more chronic and were concerned with the decision around attempting suicide. The second type of thoughts were more of an acute feeling which drove an immediate need to die. It seemed that up until this stage in the process the suicidal thoughts have been the former but as the suicidal pull becomes stronger, mothers can be at risk of developing these very acute and powerful thoughts:

*“… like an urge to do something, it’s like a really base-level urge to do something […] it’s almost like if you’re hungry or you need a wee or something you just can’t ignore that’s there and feels like it’s coming from your body rather than directly from your mind”.* (Pregnant and postpartum mother 2).

We named this acute period of suicidal ideation and desire “the darkness descends” and it involves three stages that lead to a suicide attempt: a trigger to activate this feeling, a temporary reprieve from the conflict of whether to attempt or not (in favour of attempting), and the opportunity. Mothers described the rapid onset of this “darkness”: “*it was very dark very quick”* (Pregnant mother 1) and how they stopped thinking about the positives in their life that could reduce the suicidal desire:

*“You just completely black out, you just feel like you’ve got no feelings towards anything, which is weird because actually the feelings lead you to feel suicidal but at the same time you feel like, you forget people around you, you forget the environment you’re in, you forget everything you’ve got, you know, I completely forget about my son, my family, my partner, my actually good nice life, my nice house, my job, everything, just everything is gone, just all you’ve got in your head is that you want to die”.* (Postpartum mother 2).

It appeared that mothers only needed to descend into this period of “darkness” once for a suicide attempt to be possible. However, mothers could exit this “darkness” if the attempt was not fatal, or if the conditions were not met to make an attempt - for example, if there was no opportunity or the conflict of wanting to live and wanting to die returned.

### A trigger

Mothers described that a trigger would be needed in combination with the pre-existing, more chronic suicidal ideation, to prompt “the darkness descending”. Once the suicidal thoughts became a very real possibility and women perceived a suicide attempt as a necessity, a triggering event was experienced. It also appeared the trigger occurred at a key time, rather than repeatedly, and we speculate that this is when the mother is feeling particularly vulnerable and the feelings described previously (i.e., attacked, unable, self-evaluating negatively and trapped/defeated) are particularly strong.

*“I think it’s like a trigger, like she’d have to be feeling, you know, suicidal but then something like another factor would have to come playing to sort of push her over the edge”.* (Pregnant and postpartum mother 3).

This trigger could be a trivial event to an observer, but for the mother a very small event on top of feeling attacked, and the subsequent cascade of negative thoughts and feelings, was enough to confirm that she could not give the baby a good life and/or was a “bad mother”:

*“It can be something really little, it could be something like, you know, one of the kids has hurt themselves […] you’ve not managed to get the tea on the table that night and you can just get to the point where you think ‘well I may as well not be here’. It wouldn’t take much really”.* (Postpartum mother 4).

It is important to highlight that mothers felt this trigger did not have significant meaning which suggests that mothers’ vulnerability appeared to be very high at this point and that the build-up of negative thoughts and feelings was very powerful.

### No more conflict

Women described how they stopped caring about the things in their life that prevented them from attempting suicide which provided a lapse in the previous conflicting feelings they felt and, consequently, the pull to attempt suicide became overpowering. The baby was a major concern for mothers when contemplating suicide; however, this worry seemed to vanish when the “darkness descended” and they described no longer caring about the baby:

*“I felt so suicidal that I was carrying [baby] in tears to the nurse and I just was going to give him to them and I was just looking for a way to do something to myself because I just couldn’t cope, and that was probably the worst feeling for me ever because I felt like there was no connection between me and [baby] […] I felt like I didn’t almost care about him, like, I just felt I just care about how I feel in that moment, and I just wanted to die”.* (Postpartum mother 2).

We theorised that uncomfortable feelings towards the baby contributed to prompting this overall process, and this quotation suggests that these uncomfortable feelings can also provide one of the final forces that push a mother to end her life.

There was also concern with regards to the possibility that the attempt would not have a fatal outcome:

*“You take tablets and it don’t work, then you’ve got the shame of everybody knowing what you’ve tried to do. And obviously then you’ve got to live with people knowing that you’ve attempted”.* (Postpartum mother 6).

This anxiety around whether the attempt would be lethal may be worse for mothers because the perinatal period is perceived by society as a time of happiness whereby mothers are supposed to feel such a bond with their baby that that should be enough to keep them safe. Therefore, there is also the fear of judgement from others, in the event of a non-fatal outcome. The suicide attempt is seen as the only way of escaping feelings of failure and the associated shame and isolation, but ironically these could all be amplified because of a non-fatal suicide attempt. Reaching a place where the anxiety around the outcome of the suicide attempt is diminished or overpowered again, seems to be essential for allowing mothers to attempt suicide.

### Opportunity

The final stage in our theorised process is the opportunity for a suicide attempt, and mothers outlined a number of practical conditions which would need to be met in order to act on her thoughts. Most of our sample did not have a solid plan for what sort of means they would use to attempt suicide; however, access to means is essential to attempt suicide. Therefore, the lack of plans for this aspect of the attempt may be a feature of our sample (of which the majority never attempted suicide) or it may be that the decision around suicide means is more dependent on what is available when the “darkness descends”.

A major concern for mothers when thinking about the attempt was the whereabouts of her baby while attempting and if she died: *“I know 100% I wouldn’t do it if [baby] was there because I can’t think about anything worse than me being dead and [baby] screaming his lungs out all day”* (Postpartum mother 2). Many participants described how they would need to ensure their baby was safe while they acted on the suicidal thoughts and after they died. For example, mothers described how they needed to know somebody would be coming to the home shortly after the suicide attempt so the baby would not be left alone for a long period of time, or the baby would need to be in somebody else’s care while they made a suicide attempt.

As well as access to means, we were also interested in how participants would choose which means to use to attempt suicide. It became apparent that participants considered different means of suicide carefully and ensuring a fatal outcome was very important:

*“I didn’t want to do anything that was going to be with a high fail rate. So, you can try and overdose but that might not work but if I hang myself, you know, there’s no way out of that once you start and I think it’s the most common method for guys, so I thought ‘tried and tested method’”.* (Pregnant mother 1).

Participants described how they would choose a more violent method of suicide because a drug overdose may not be lethal. We described mothers considering the possibility of a non-fatal suicide attempt in *‘6.2 No more conflict’*, and we theorised mothers are more motivated to choose a violent method to ensure escape but also to ensure she would die and not have to live with the repercussions of surviving which were perceived as worse than death.

Overall, our model outlines a process whereby mothers experience feeling attacked by changes brought about by the perinatal period. This initial attack then prompts a cascade of increasingly negative thoughts and feelings, including feeling unable to cope, failure, negative self-evaluation, and entrapment. Once mothers conclude that the only option to solve their entrapment and benefit others is suicide, they are at risk of the “darkness descending”. If both the mental and environmental conditions are met once the “darkness descends”, mothers can act on their thoughts of suicide.

## Discussion

We aimed to develop a grounded theory model outlining what makes a woman suicidal during the perinatal period. To the authors’ knowledge, this qualitative investigation is the first to use a grounded theory approach to gather and analyse interview data investigating suicidal thoughts and behaviour irrespective of diagnosis, during pregnancy and the first postpartum year. We not only theorised how a mother’s suicidal ideation might develop but also what might move a mother to act on her thoughts and engage in suicidal behaviour.

Investigating the psychological underpinnings of suicide during the perinatal period poses a particular challenge because the transition to motherhood or becoming a mother again is a psychological phenomenon, per se. How women experience, perceive, and make sense of this transition has been extensively researched. For example, Nelson [38] conducted a meta-synthesis of nine qualitative studies that explored maternal transition in North America and Australia. Nelson [38] found two processes that were active during maternal transition: engagement, and growth and transformation. Although, these concepts were not involved in the current grounded theory findings, a malfunction of the transition to motherhood may contribute to the development of suicidal ideation. An unwillingness or inability to engage in the transition to motherhood, for example if the mother is not sure whether she wants to continue the pregnancy or feels out of control of her environment, could contribute to the initial feeling of being attacked by motherhood. Similarly, participants described feeling trapped and defeated which is in stark contrast to the concepts of growth and transformation that Nelson [38] reports.

It is also important to compare our findings with investigations of maternal mental health difficulties that did not focus on suicidal ideation and behaviour, to consider how our model differs. Homewood et al. [39] conducted a grounded theory of mothers with postnatal depression and theorised that these mothers became occluded by the overwhelming responsibility for their infant’s needs, trying to meet their infant’s needs and negative self-evaluation. However, mothers then re-emerged from the distress (and occlusion) by distancing themselves from their children’s dependency through accepting their ambivalent feelings and readjusting their boundaries with their infants. This is interesting because our theorised stages of feeling attacked by motherhood, feelings of failure and the inability to cope and negative self-evaluation, are similar stages to those outlined by Homewood et al. [39]. However, we found our participants did not re-emerge from the distress but instead descended into entrapment and defeat which led to suicidal ideation. This leads us to speculate that perhaps the difference between mothers who are depressed but not suicidal and mothers who are suicidal, is the ability, or inability, to distance themselves from their infant’s dependency, whether that be through the independence weaning can bring or better social support.

The *three-step theory of suicide* [11] proposes that a combination of being in pain and hopelessness leads to suicidal ideation. Our model, although similar, is more specific in that it identifies feeling attacked by motherhood (which could be interpreted as the cause of pain) and triggering the process culminating in suicidal ideation and behaviour. However, we did not find that participants described hopelessness explicitly, rather the finality and endlessness of having a baby was emphasised, leading to mothers feeling trapped or defeated. Praetorius, Maxwell and Alam [18] built on Joiner’s [8] *ITS* in which they describe ‘mommy failure’, baby burden and thwarted motherhood as additional elements, specific to mothers with postpartum depression, that contribute to the feelings of perceived burdensomeness, thwarted belongingness and capability that result in suicidal behaviour. Praetorius, Maxwell and Alam [18] do not mention the crucial element of entrapment and defeat that we found to be so important to mothers in the current study. Moreover, we found mothers experienced intense conflict when considering attempting suicide and that they needed to be relieved of this conflict in order to act on their thoughts; this important stage does not feature in Praetorius and Alam’s [18] augmented *ITS* model.

Similarly to our model, O’Connor’s [10] *IMV* identifies entrapment and defeat as major drivers in suicidal ideation formation, during the motivational phase. We also identify coping as a factor that can contribute to someone feeling trapped, similarly to the *IMV*. Interestingly, impulsivity is given as an example of a volitional moderator in the *IMV*, which can move someone from experiencing suicidal ideation to acting on their thoughts. We found mothers would thoroughly consider the consequences of dying by suicide before attempting, and that both a lapse in the conflict around deciding to attempt and the opportunity to attempt needing to be present, enabled a mother to attempt. Therefore, ‘impulsivity’ seems to overly simplify the specific conditions required to attempt suicide, that we identified. This lack of impulsivity may only apply to this sample or mothers, who have strong intentions at the point where a suicide attempt becomes a possibility. Other populations may have lower intentions to die but the opportunity is more readily available and therefore impulsivity is a major driver for an attempt, for example those in rural China [40]. Alcohol and substance use can be used to escape emotional pain but also promote impulsivity. None of the participants described alcohol or substance misuse as a precursor to their suicidal thoughts and this may also explain why impulsivity did not seem to factor in this study.

Interestingly, we also found participants described two types of suicidal thinking: 1) those that were more chronic and were concerned with weighing up suicide as an option, and 2) the acute thoughts which brought an immediate need to die. A recent study of 6200 adults in the United States of America investigated the relationship between suicidal thought content and suicidal behaviour, separating suicidal thoughts into those that are passive (e.g., “I wish I could disappear”) and those that are active (e.g., “I should kill myself”) [41]. Findings showed that having passive thoughts alone, active thoughts alone and combined passive and active thoughts, were all significantly associated with lifetime and past-month suicide attempts. More interestingly, the authors also reported that individuals who attempted suicide most frequently experienced passive suicidal ideation only, which was assumed to be less likely to enable a person to act on their thoughts than active suicidal ideation. A recent meta-analysis also found that passive suicidal thoughts are highly prevalent and strongly associated with suicide attempts and death [42]. Although we found our sample described both passive and active suicidal thoughts, it was the thoughts concerned with weighing up the decision whether to attempt, and the very “dark”, intense thoughts whereby mothers felt a need to attempt, that we found to be the most important types of thoughts in mothers’ narratives. As far as we are aware thought content similar to that found in our study has not been described elsewhere in the suicide literature and this is an important and novel finding.

### Strengths and limitations

This novel qualitative investigation is the first to focus specifically on suicidal ideation and behaviour, irrespective of mental health diagnosis, during the perinatal period. A strength of this study is the recruitment of participants across the UK, rather than from one locality. Recruitment of mothers who feel/have felt suicidal is particularly challenging because feeling suicidal during the perinatal period is incredibly stigmatised (many of the participants had never told their family how they felt) and poses ethical difficulties. Therefore, achieving a sample size of 12 perinatal mothers and reaching theoretical sufficiency are strengths of this study.

We need to acknowledge that our sample was limited in that very few mothers that we interviewed had attempted suicide. Therefore, the part of our model that outlines how a mother transitions from suicidal ideation to attempting suicide was mostly developed through mothers describing this transition hypothetically. The sample was not as diverse as we had planned for with regards to age, ethnicity and educational attainment, and we do not know how diverse participants were in terms of socio-economic status. We had also hoped to recruit more women who were experiencing suicidal thoughts/behaviour during the perinatal period at the time of the interview to gather more raw narratives of how mothers experience this time during crisis.

We also feel it is important to highlight that, as this study focused on the experiences of mothers in the UK, this is a Eurocentric investigation. Although this is not necessarily a limitation of this study, because we did not aim to gather experiences of mothers internationally, some of the concepts described in our model may not be recognisable by women in other countries and communities.

### Implications and future research

The speed at which mothers became suicidal varied but some participants highlighted that the process could be very rapid. Therefore, identifying when women are experiencing the cascade of feelings and thoughts outlined in our model is of paramount importance to prevent and reduce suicidal thoughts and behaviour. Continuity of care during the perinatal period is vital to facilitate a trusting relationship between mother and health professional which may encourage disclosure of suicidal thoughts and experiences potentially leading to a suicidal crisis. To facilitate identification at routine appointments, midwives and health visitors need to ask questions that investigate the factors we theorise contribute to a mother feeling attacked, such as her feelings towards the baby and isolation, as well as enquiring about how she views herself, entrapment, and defeat. Professionals should also prioritise reassuring mothers that disclosure of suicidal thoughts helps professionals identify appropriate support and will not necessarily lead to a social services referral. Although none of the participants were ever told they were failing in their mothering abilities, some mothers described feeling criticised by health professionals and this perpetuated their negative thoughts and self-evaluation. It is important for health professionals working with mothers during the perinatal period to remember women can be extremely sensitive to anything that could be interpreted as judgement or criticism, and that maternity professionals’ advice should aim to encourage and boost confidence. With regards to interventions for preventing and reducing suicidal thoughts, improving antenatal education to include psychoeducation about the transition to parenthood, a focus on helping women to become more aware of and manage their own, and others’, expectations of pregnancy and motherhood, and identify social support contacts, would be a useful public health strategy for improving the experiences and mental wellbeing of all mothers. Helping women investigate and challenge their feelings of failure when mothering and the origins of their negative self-evaluation would also be useful directions for tailoring existing talking therapies to mothers experiencing suicidal thoughts during the perinatal period.

Future research needs to involve the longitudinal quantitative measurement of the stages outlined by our model to 1) triangulate our qualitative findings, 2) investigate the time period this process can take more closely and how much this varies between mothers, and 3) provide more evidence of what differences there are between those that do not experience suicidal thoughts, those that experience suicidal thoughts only and those that go on to attempt suicide. Qualitatively investigating the experiences of health professionals who work with suicidal women during the perinatal period would provide a more holistic picture of how suicidal thoughts are triggered and develop in this population, when combined with the current findings.

## Conclusion

This grounded theory has identified a cascade of thoughts and feelings experienced by mothers that can culminate in suicidal thoughts and behaviour during the perinatal period. Participants stressed the rapid onset of suicidal thoughts; this adds to the importance of health professionals investigating the thoughts and feelings outlined in our model at routine appointments, for prevention and reduction of suicidal thoughts and behaviour.

## Data Availability

The datasets generated and analysed during the current study are not publicly available due to the sensitive nature of the interviews but are available to bona fide researchers from the corresponding author on reasonable request.
